# Biological analysis of cancer specific microRNAs on function modeling in osteosarcoma

**DOI:** 10.1038/s41598-017-05819-7

**Published:** 2017-07-14

**Authors:** Hao Wang, Min Tang, Liping Ou, Mengyi Hou, Tianyu Feng, Yu-E Huang, Yaqian Jin, Heng Zhang, Guowei Zuo

**Affiliations:** 1 Key Laboratory of Diagnostic Medicine designated by the Chinese Ministry of Education, Department of Laboratory Medicine, Chongqing Medical University, Yuzhong district, Chongqing 400016 China; 2 Department of Urology, Northwestern University Feinberg School of Medicine, Chicago, Illinois 60611 USA

## Abstract

Osteosarcoma (OS) is the most common bone tumor characterized with a high risk of amputation and malignant morbidity among teenagers and adolescents. However, relevant pathogenic/biological mechanisms underlying OS-genesis remains to be ambiguous. The aim of this study was to elucidate functional relationship about microRNAs-mRNAs networks and to identify potential molecular markers via a computational method. Gene expression profile (GSE70415) was recruited from Gene Expression Omnibus. 3856 differentially expressed genes and 250 significantly expressed microRNAs were identified by using GCBI. The results of GO and KEGG pathways associated proteomics analysis indicated that extracellular matrix organization, small molecule metabolic process, cell adhesion (GO IDs: 0030198, 0044281, 0007155) and pathways in cancer, PI3K-Akt signaling pathway, metabolic pathways (pathway IDs: 5200, 4151, 1100) were significantly enriched. In addition, CKMT2, miR-93b-5p, miR-29b-3p were found to be positively/negatively correlated with TP53, EGFR, and MMP members mediated OS development, including angiogenesis, migration and invasion. Further visualization of collective effect of 1181 microRNAs-mRNAs pairs and protein-protein interactions was realized by applying with cytosacpe. In summary, our work provided a better understanding of non-coding regulatory mechanisms of transcriptomics and unraveled essential molecular biomarkers in osteosarcoma.

## Introduction

Osteosarcoma (OS) is the most frequent primary bone malignancy, characterized with a high potential for lung metastasis and has been the third common cancer-associated threat to adolescents^1^. It most occurs at the extremities of long bones, where osteoblasts transform into mature bone tissue. However, the putative molecular mechanisms underlying OS carcinogenesis have not been deciphered completely and still been a challenge yet. Hitherto, cumulative evidences^2–7^ have demonstrated that a variety of factors including microRNAs (miRNAs), a group of non-coding RNAs, were involved in OS development. The first study on miRNAs expression in OS published by Gao *et al*.^8^ identified 182 differentially expressed miRNAs (DEmiRNAs), accelerating revelation that miRNAs may have an obscure but critical impact underlying OS pathogenesis. Recent researches^9–12^ also suggested that miR-1, -409-3p, -379, -665, -489-3p function as sequence-specific tumor suppressors mediating primary OS proliferation, cell death and even distant metastasis.

Alternatively, development of high throughput testing technology (microarray, next-generation sequencing) has successfully made it convenient to acquire large-scale genetic data. Bioinformatics approach uniting biology, mathematics, and computer science has further widely facilitated molecular mechanism explanation and discovery of tumor-correlated diagnostic markers. RNA-sequencing^13^ has found that amounts of genes come into discrepancy along the course of bone malignancy transformation. By comparing mRNA expression profiles between OS tissues and cell lines and xenografts, Kuijjer M L *et al*.^14^ initially achieved histological subtyping classification (osteoblastic, chondroblastic, fibroblastic) at transcriptome level. In parallel, epigenetic events and RUNX2 interactome were identified to be constitutively activated in OS^15^.

Nevertheless, targeting networks of miRNAs to mRNAs underlying osteosarcomagenesis have not been systematically interpreted yet. MiRNAs are essential components in biological homeostasis and the current paper has accomplished miRNAs-involved networks construction and exploitation for essential biomarkers along pipeline of central dogma by means of easy-handling web-based analytical tool GCBI.

## Results

### DEGs and DEmiRNAs between hMSC and OS cell lines

**Figure 1:**
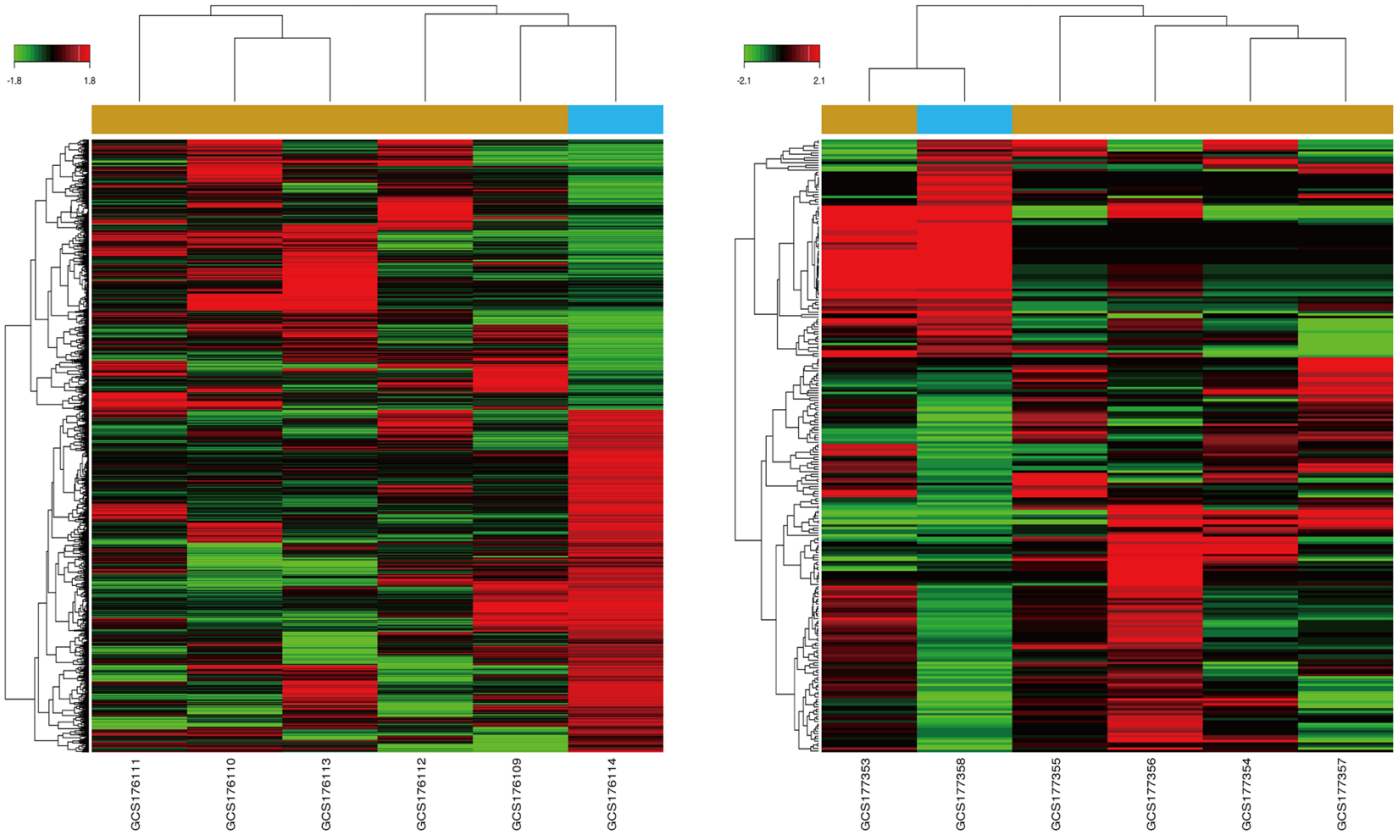
Profiles of differentially expressed genes and miRNAs in osteosarcoma cells. Data of both mRNA (a, left) and miRNA (b, right) were clustered using GCBI (P < 0.01). In total, 3856 DEGs and 250 DEmiRNAs were deprived comparing to control group (blue).

The sample set GSE70415, which consists of miRNA (GSE70367) and mRNA (GSE70414) expression profiles of five human OS cell lines (MG63, Saos, HOS, NY, Hu09) and a corresponding control (hMSC) was obtained from Gene Expression Omnibus (GEO). Following standard protocol^16^ of samples qualification and normalization, raw expression values were summarized and analyzed in a consecutive workflow (seen in Fig. S1) based on GCBI. In total, 3856 (P < 0.01) significant DEGs were identified, of which 1705 over-presented and 2151 showed an attenuated behavior (Fig. 1a). Periostin (POSTN), a canonical osteoblast marker, has not only exhibited a most significant declination among the whole collection, but recent study has also already verified hypothesis that aberrant stimulation of it concerned with bevacizumab induced resistance in the cases of glioma implementing with anti-VEGF-A therapy^17^. Meanwhile 250 (P < 0.01) DEmiRNA picked out from microRNA repertoire comprised by 161 ascent items and 89 down-regulated miRNA episodes (Fig. 1b). Whereas, of some limitation, miR-182-5p and miR-708-5p, existing the highest contradictory deviation (absolute fold change|FC > 100) within current community events both could not be tracked among 81 small sequences in curated Osteosarcoma Database^5^. Along the clarification of microRNA-engaged epigenetic reprogramming, potential connection between both of them and vorinostat, an approved histone deacetylase inhibitor was further validated in 143B and MG63 (data have not been published). In addition, DEGs which were statistically significant complied with cumulate information partially (about 7%) after matching to 911 trustworthy entries within the Osteosarcoma Database (seen in Fig. S2). The full tables of DEGs and DEmiRNAs were included in Tables S2 and S3.

### Functional enrichment of DEGs and DEmiRNAs between hMSC and OS cell lines

**Figure 2:**
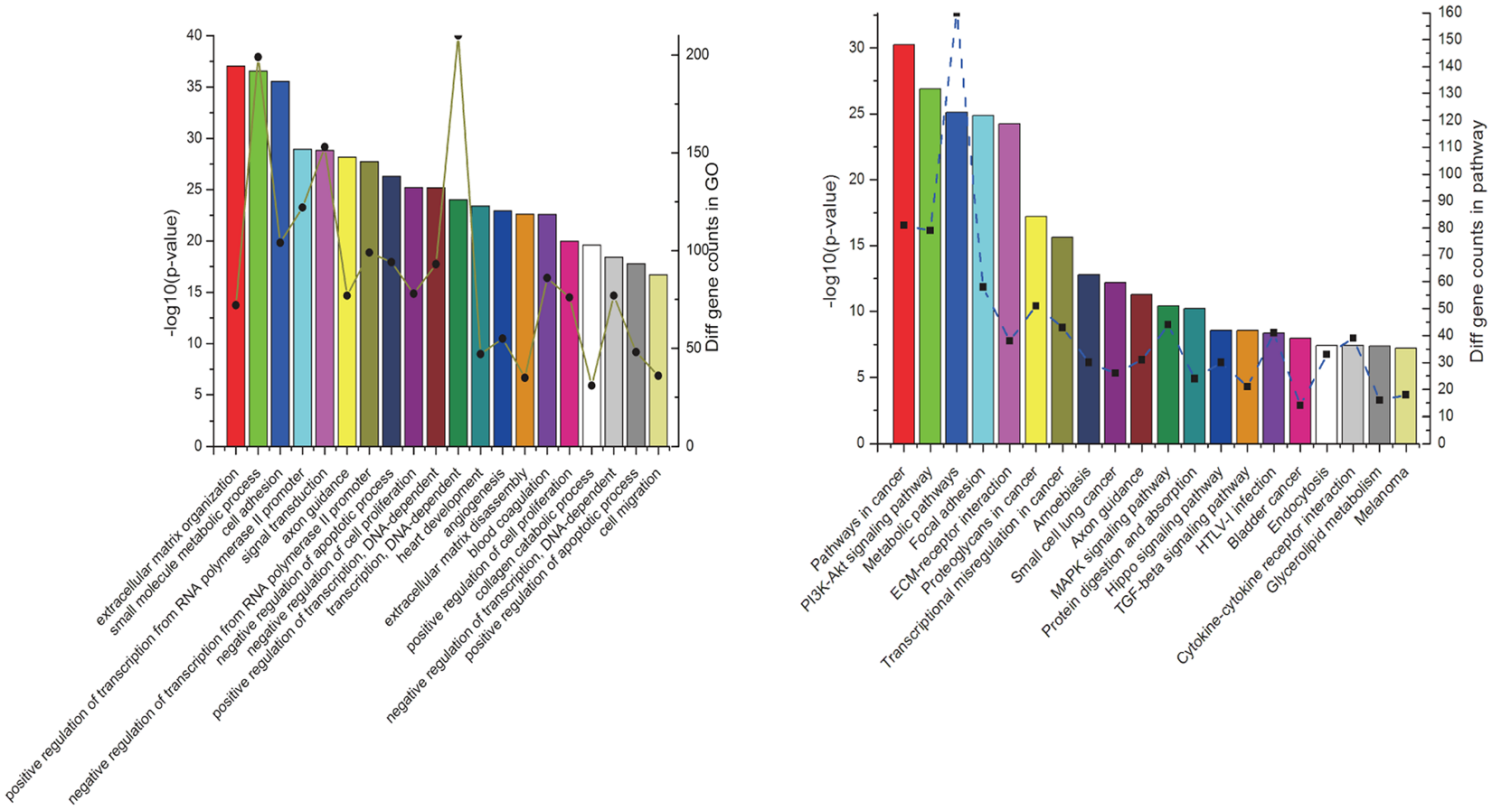
Representative GO and KEGG pathways enrichment analysis of osteosarcoma. Significantly changed GO (a, left) and KEGG pathways (b, right) of predicted DEGs were illustrated. The left y-axis titled with −log_10_P and the right y-axis presented DEGs while the x-axis showed GO/KEGG category. The larger −log_10_P indicated a smaller P-value. −log_10_P: negative logarithm of the P value.

**Figure 3:**
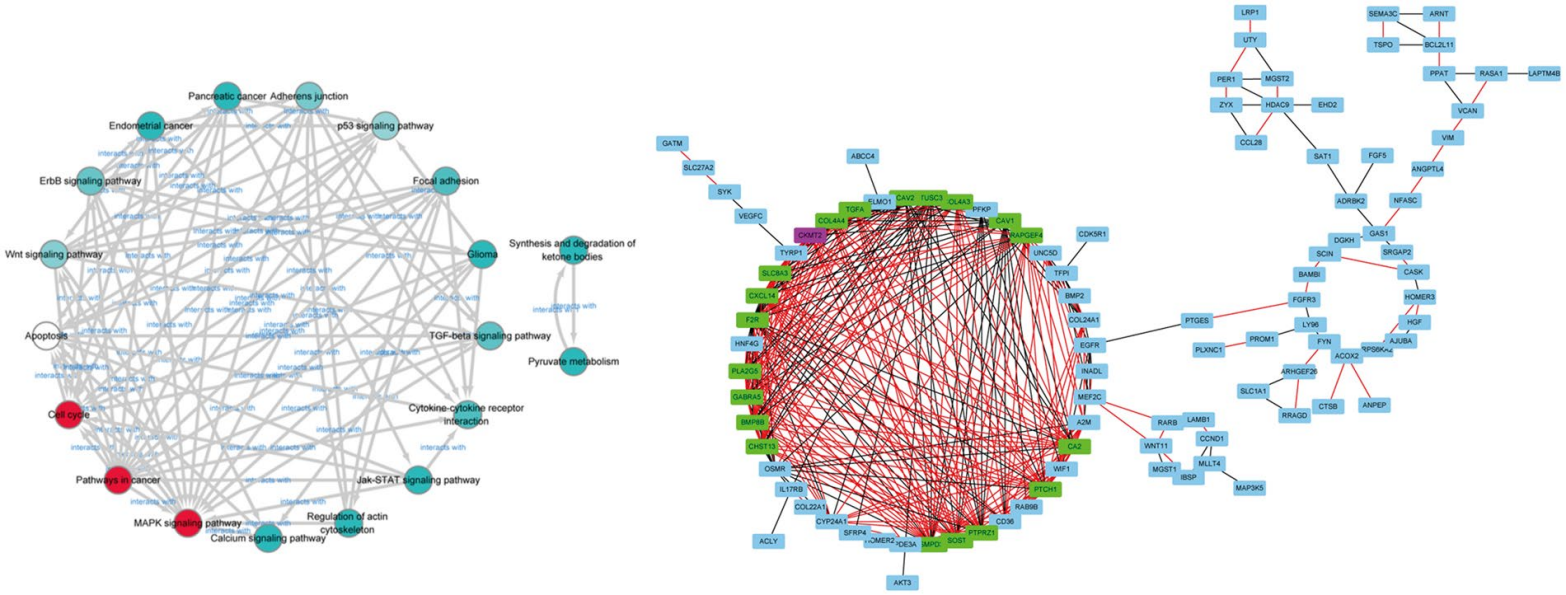
Co-expression network analysis of osteosarcoma. Significantly coefficient KEGG pathway network (a, left) was visualized with augmented index degrees (circles from cyan to red). Co-expressed DEGs were integrated into networks using bioinformatics methodology (b, right). Positive/negative function among common genes (rectangles, blue) and even tightly clustered elements (purple and green) were displayed with different colors (red and black).

As known, tumorigenesis is featured with a number of biological disorders and cellular events dysregulation, such as angiogenesis, cell adhesion, signaling transduction. Thus, it is absolutely necessary to unravel discrepant biological processes and pathways recruited along the duration of neoplasia. In the enrichment modules, 395 records of GO and 142 KEGG pathways (full tables can be seen in Tables S4 and S5) were verified through employment with Fisher exact testing and FDR^18^. Moreover, we annotated top-ranked 20 GO and KEGG pathways respectively without distinguishing biological process (BP), cell component (CC) and molecular function (MF) (Fig. 2a and b). It is obvious that the top three enriched biological processes contained extracellular matrix organization, small molecule metabolic process, cell adhesion (GO IDs: 0030198, 0044281, 0007155). Whereas, pathways in cancer, PI3K-Akt signaling pathway, metabolic pathways (pathway IDs: 5200, 4151, 1100) were three most significantly concentrated pathways through which oncogenes silencing was switched on or off. Both GO and KEGG pathway enrichment analysis showed a peak distribution of DEGs in metabolic dysfunction. To some extent, this was consistent with previous consensus^19^ that tumor events, such as proliferation, metastasis and angiogenesis could be partially attributed to hypermetabolic activity of neoplasm. Besides, MAPK signaling pathway, pathways in cancer, and cell cycle (pathway IDs: 4010, 5200, 4110) acted as leading initiators mediating follow-up aberrant pathway cascades through assessment of determination coefficient (Fig. 3a). Intensive pathways featured with more than 10 contribution degrees were formatted into Table 1.

**Table 1:** Remarkably correlative pathways in functional enrichment.

Pathway ID	Pathway name	Degree	Pathway feature
4010	MAPK signaling pathway	34	down|up
5200	Pathways in cancer	27	down|up
4110	Cell cycle	24	down|up
4210	Apoptosis	23	down|up
4115	p53 signaling pathway	17	down|up
4310	Wnt signaling pathway	16	up|down
4520	Adherens junction	15	down|up
4012	ErbB signaling pathway	14	down|up
4350	TGF-beta signaling pathway	13	down|up
4510	Focal adhesion	12	down|up
4060	Cytokine-cytokine receptor interaction	12	down|up

### Genes interplay and co-expression networks

To further explore and clarify realization of message or communication flow from member to member scattered at the crossing pathways, visualization and cluster analysis of hub genes were accomplished using cytoscape 3.4.0. We picked out 698 overlapped genes derived from GO and KEGG pathway analysis and applied them to genesignal (shown in Fig. S4) and co-expression network construction (Fig. 3b). As co-expression graphic illustrated, correlative genes positively or negatively interacted with their neighbors in a non-direction nested manner. According to MCODE^20^ analyzer, 19 subordinated nodes intimately clustered to creatine kinase, mitochondrial 2 (CKMT2), also known as SMTCK, which was indispensible when maintaining rational energy metabolism. Thus, our colleague later validated hypothesis that CKMT2 might as a key regulating factor participating in osteosarcomagenesis (data have not been published).

### Targets prediction and miRNAs-targets interaction

**Figure 4:**
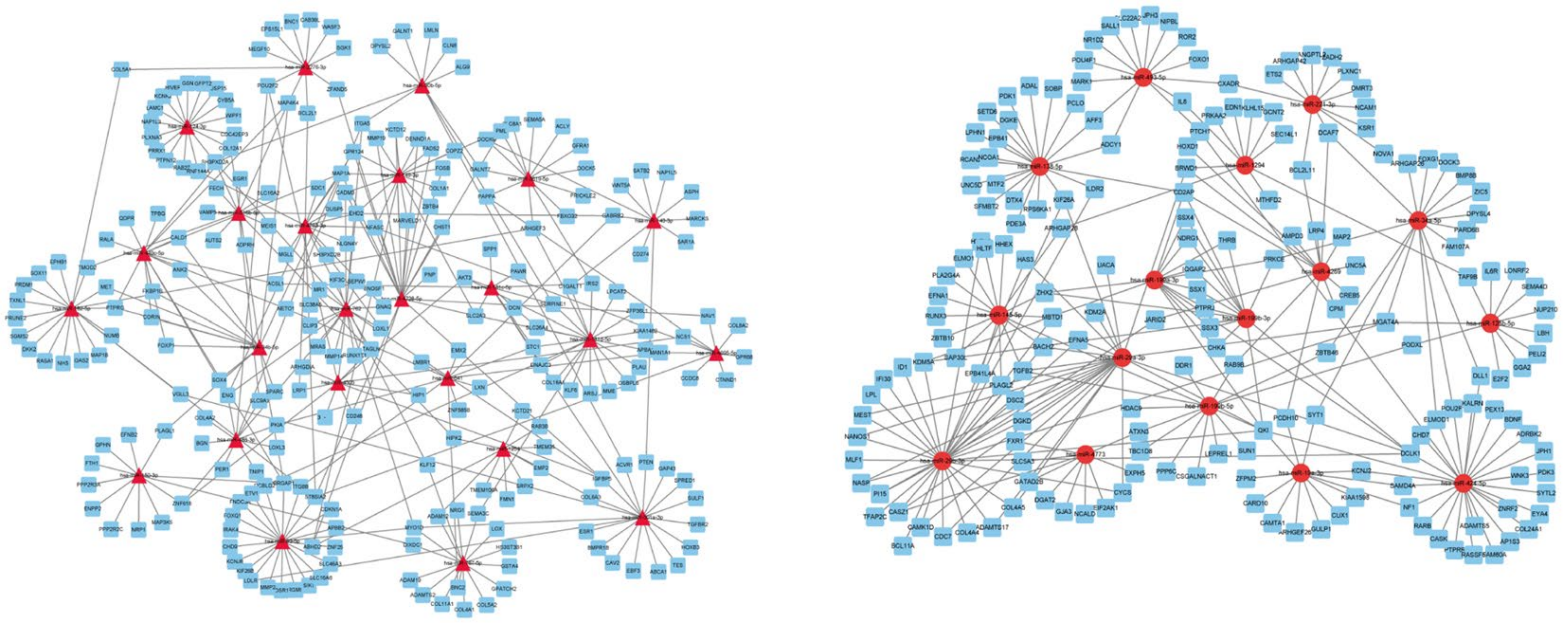
Regulatory networks of onco-associated miRNAs in osteosarcoma cells. Functional models of up-regulated miRNAs (a, left) and down-regulated miRNAs (b, right) were constructed, respectively. Up/down-regulated miRNAs were exemplified with triangles or circles with red color whereas targeted genes showed with blue squares.

MiRNAs, a group of well-known endogenous non-coding RNA, usually act as transcription regulators during gene expression through binding to 3′-untranslated region (3′-UTR) of target mRNAs. It is explicit that diversity of miRNAs resulted from length or alignment of seed region complicates regulatory models. Thus, further understanding net-association between miRNAs and mRNAs is extremely needed. By utilizing GCBI that integrating TargetScan^21^ and miRanda^22^ databases, we mined out 250 DEmiRNAs with an up to down ratio at 161/89 (shown in Fig. 1b). Abiding by the base-pairing principle, there were 29227 genes found deposited in the target pools (TargetScan and miRanda). Conversely, 388 were substantially involved in GO enrichment (seen in Fig. S3) and 608 were mingled with DEmiRNAs regardless of exact binding pair bases. To delineate miRNAs-mRNAs axis vividly, we postulated index degrees which changed no less than 10 to be of significance in transcription function in our research and deeply screened impaction networks of selected 40 DEmiRNAs (shown in Table S1). Illustration of connective networks of miRNAs and corresponding targets were realized using cytoscape 3.4.0. In summary, 238 downstream genes were blocked and 181 targets found to be in an activated status (Fig. 4a and b). The results showed that either lower-expressed miR-29b-3p or over-presented miR-93-5p was hub miRNA possessing most significant impact on gene transcription and even protein function implement.

### Protein-protein interaction in OS cell lines

**Figure 5:**
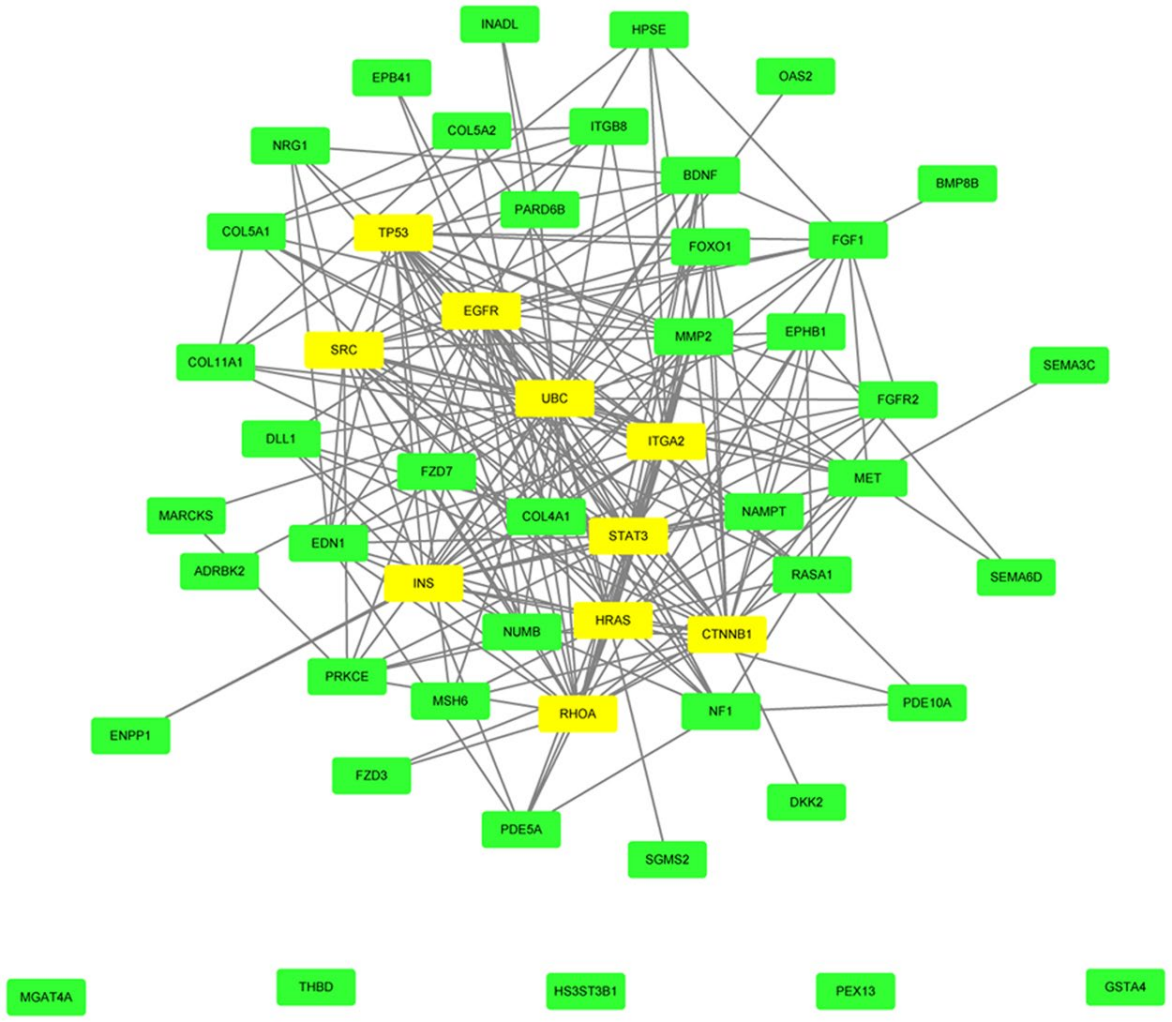
Predicted protein-protein interaction (PPI) in osteosarcoma. Bioinformatics prediction of significantly involved proteins (green) in our experiment and essential proteins in databases (yellow).

To study protein-protein interactive association of DEGs mediated by DEmiRs, we screened 35 typical DEmiRs (FC ≥ 10 compared to control) and integrated protein-protein interaction (PPI) network of under-manipulated target mRNAs by means of STRING 10.0. Neither six isolated nodes (has-miR-941, 127-3p, 487b-3p, 34a-3p, 493-3p, 654-3p) without microRNA-mRNA joint nor molecules that absent from function (GO or KEGG) participation was eliminated. Then emerged 43 genes were employed to construct PPI network by using cytoscape 3.4.0 (Fig. 5a). Within shaped model, receptor nodes already have been verified or not, such as FOXO1, BMP, members of COL and ITG families were predicted to interact with members essential for pathway perturbation, among which some classical suppressive factors involved, like TP53, EGFR and MMP2.

## Discussion

In this study, we firstly provided a systematical miRNAs-mRNAs functional model based on expression profiles of OS transcriptome. Distinctive to previous researches focusing on individual command element, we analyzed a large number of molecules and integrated them into a functional network via adopting a bioinformatic approach. This research is not only a promotion in revealing small non-coding RNA disorder hiding in oncogenesis, even chemoresistance, but also indispensable for clinical early-screening and targeted therapy exploitation^23^, though underveining disturbance mediated by genetic or microenvironment origin remains to be a challenge.

By microarray analysis, we firstly identified 3856 mRNAs and 250 miRNAs which significantly diverged in OS cells. POSTN, mainly involved in osteoblasts adhesion and differentiation^24, 25^, was found declined remarkably in OS subgroups comparing to normal sets. Nevertheless, what fascinated us was that expression of POSTN had been reported to remain at a high level in OS compared to osteochondroma and high content of POSTN intensely correlated with tumor angiogenesis and poor prognosis in the OS as well as high grade glioma *in vivo* ^17, 26, 27^. The probable reasons for this discrepancy might be inconsistent of sample type (cell lines versus specimens)﻿ and detection approaches (RNA microarray versus immunohistochemistry). Subsequently, the results of functional enrichment analysis demonstrated that metabolic pathway played an important role and a large number of cancer associated pathways were distinguished, including PI3K-Akt and MAPK signaling. There is a reason to believe that chemoresistance is relevant to metabolic abnormality as miR-221, −101, −22, −155^28–31^ have already been proved to participate in cisplatin and doxorubicin derived chemo-resistance as well as our investigation about SAHA to miR-182-5p and -708-5p in OS cells. Alternatively, activation of the PI3K-Akt pathway suppressed cell longevity through phosphorylation of FOXO members and balancing its activity with MAPK and NF-κB pathway intimately associated with tumors survival^1^. On the other hand, stimulation of MAPK signaling was confirmed to link with elevated EGFR phosphorylation and MMP-9 levels mediated by lowering miR-143 in OS^32^. Except those miRNAs-pathways^5, 7, 33^ verified so far, newly discovered miRNAs expanded OS related miRNAs spectrum notably. Furthermore, modeling of miRNAs-mRNAs networks was achieved using a well-established tool to visualize intricate nodes connections (Fig. 4a and b). Despite not the most altered, miR-29b-3p and miR-93-5p were two core upstream elements targeting transcription proceeding of which miR-29b-3p induced OS depression had been affirmed to be with tumor-specific subcellular localization^34^ while the most significant miR-182-5p and miR-708-5p displayed relatively moderate and even lower contribution degrees. It seems that efficiency of miRNAs is not simply determined by the level of variation but relied on critical GO and pathways. In summary, 1181 linkages have been established in the current study, which has been a striking acceleration about non-coding unit mediated OS carcinogenesis.

Bioinformatics approach combining GCBI and cytoscape, an innovative pipeline distinguished from troublesome data processing to pattern display, facilitating dimensional molecular interaction and model analysis based on antecedent data and improved algorithm multidisciplinary. It is insufficient that our present paper has just successfully explained the relationship between microRNAs and coding targets, and necessary to further supplement another non-coding factors, including long non-coding RNAs, circle RNAs mediated competitive mechanism. There is no doubt that combinational strategies through employing identification of group effect of non-coding RNAs-mRNAs-proteins even small inhibitors and drugs would be potent approaches and might bring a breakthrough.

## Methods

### Data source

In the present paper, gene expression profile (accession: GSE70415) deposited in the Gene Expression Omnibus (GEO)^35^ (https://www.ncbi.nlm.nih.gov/geo/) was utilized. GSE70415 comprised a total of five OS samples (MG63, Saos, HOS, NY, Hu09) and a normal control (hMSC). Subset expression profiles of mRNAs (GSE70414) and miRNAs (GSE70367) were detected using Affymetrix Human Genome U133 Plus 2.0 Array and Affymetrix Multispecies miRNA-3 Array (Inc. Santa Clara, CA, USA) based on platform GPL 570 and 16384. Totally, 54675 genes and 25533 miRNAs were mined, respectively.

### Differentially expressed genes and miRNAs-mRNAs analysis

To identify DEGs and DEmiRNAs between OS and hMSC cell lines, a web-based online tool GCBI (www.gcbi.com.cn/gclib/html/index) was utilized. Entries qualification and calibration were then achieved by taking standard Median Polish algorithm^16^. Only probe signals with p-values < 0.01, false discovery rate (FDR) < 0.01 and absolute value of fold change (FC) > 2 were considered to be statistically differential. Genesignal and co-expression network were further constructed based on contribution degrees according GCBI protocol (http://college.gcbi.com.cn/helpme).

### Enrichment analysis and networks construction

For visualization, cytoscape 3.4.0^36^ (http://www.cytoscape.org/), an open source platform, was utilized to portray the relationship among target molecules. Gene Ontology (GO) and the Kyoto Encyclopedia of Genes and Genomes (KEGG) pathway enrichment analysis^18^ for DEGs was performed with GCBI mentioned above. DEGs and DEmiRNAs given previously were selected out to construct networks. Molecular Complex Detection (MCODE)^20^, based on vertex weighting by local neighborhood density and outward traversal from a local dense seed to the isolate the dense regions, was employed to find molecular complexes.

### Databases

To identify putative regulatory correlation within our work, some essential databases were concentrated to explore the relevance of non-coding transcriptome to proteomics. STRING10.0^37^ (http://string-db.org/) was implemented to supervise protein-protein interactions with. MiRNAs which associated with oncogenesis were further filtered according to osteosarcoma^5^ (http://osteosarcoma-db.uni-muenster.de/) and oncomiRDB^33^ (http://bioinfo.au.tsinghua.edu.cn/member/jgu/oncomirdb/).

## Electronic supplementary material

Supplementary Information

Table S2

Table S3

Table S4

Table S5
