# Supplementary material for: Biological analysis of cancer specific microRNAs on function modeling in osteosarcoma
Source: Sci Rep. 2017 Jul 14;7:5382. doi: 10.1038/s41598-017-05819-7 (PMC5511279; doi:10.1038/s41598-017-05819-7)
Supplement: Supplementary file 1 — Supplementary Information [file 41598_2017_5819_MOESM1_ESM.pdf]

Biological analysis of cancer specific microRNAs on function modeling in osteosarcoma

Hao Wang[1#], Min Tang[1#], Liping Ou[1], Mengyi Hou[1], Tianyu Feng[1], Yu-E Huang[1], Yaqian Jin[1], Heng Zhang[2], and Guowei Zuo[1]*

[1]Key Laboratory of Diagnostic Medicine designated by the Chinese Ministry of Education, Department of Laboratory Medicine, Chongqing Medical University, Yuzhong district, Chongqing, 400016, P. R. China

[2]Department of Urology, Northwestern University Feinberg School of Medicine, Chicago, Illinois, 60611, USA;

E-mail address: elison.wang@qq.com (H. Wang); catom@126.com (M. Tang); olp1979@163.com (L. Ou); hmyi794@vip.qq.com (M. Hou); tianyuFeng0408@qq.com (T. Feng); 156464331@qq.com (E. Hang); 710337704@qq.com (Q. Jin); Hzhzhan@gmail.com (H. Zhang); gwzuo@qq.com (G. Zuo)

*Corresponding author. Tel.:+86 18725697317; Fax: +86 023-68485240; E-mail: gwzuo@qq.com

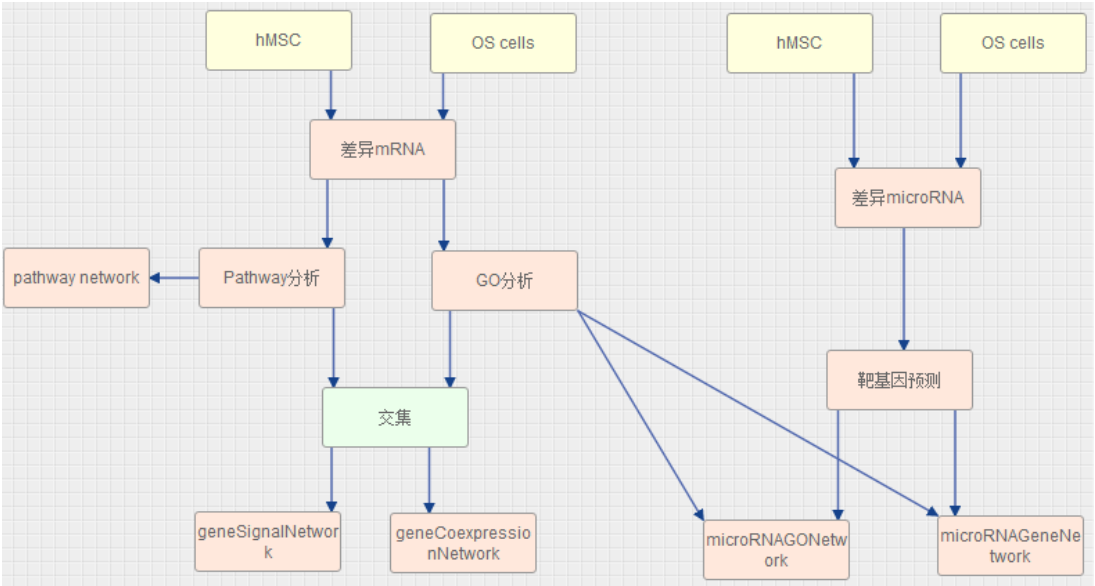

Fig. S1 Biological workflow of osteosarcoma associated non-coding regulatory pattern.

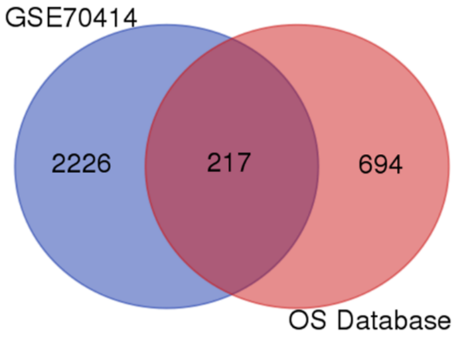

Fig S2 Differential genes matching within Osteosarcoma Database.

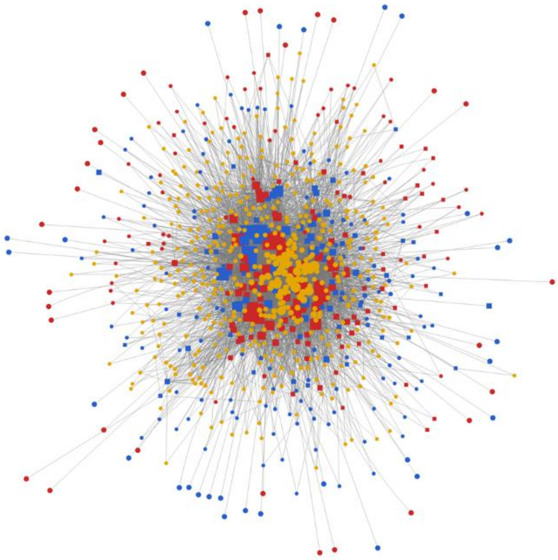

Fig. S3 MiRNAs-GOs-network of osteosarcoma. According to bioinformatics methodology, miRNAs- GOs interaction was constructed to illustrate the key regulatory function. The squares represent up/down-regulated (red, blue) miRNAs, while solid circles represent corresponding GOs. The bigger size of squares or circles means the higher degrees.

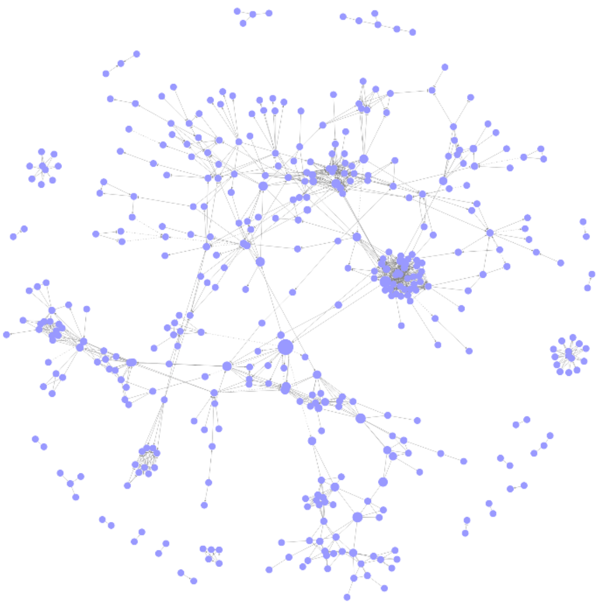

Fig. S4 genesignal network of osteosarcoma. Genes-genes network derived from proteins-proteins interaction according to GO and KEGG by using GCBI platform. The purple solid circles represent genes and the size represents betweenness centrality, and lines between two nodes mean correlative relationship.

Table S1 40 canonical DEmiRNAs in mRNAs transcriptional networks of osteosarcoma

Transcription ID	Biotype	miRNA feature	Degree	Rank
has-miR-93-5p	miRNA	up	22	4
has-miR-181d-5p	miRNA	up	21	6
has-miR-4728-5p	miRNA	up	21	7
has-miR-182-5p	miRNA	up	19	8
has-miR-301a-5p	miRNA	up	19	9
has-miR-762	miRNA	up	19	10
has-miR-124-3p	miRNA	up	17	11
has-miR-149-3p	miRNA	up	17	12
has-miR-4763-3p	miRNA	up	17	13
has-miR-449c-5p	miRNA	up	16	16
has-miR-767-5p	miRNA	up	16	17
has-miR-34b-5p	miRNA	up	14	20
has-miR-3619-5p	miRNA	up	13	24

has-miR-516b-5p	miRNA	up	13	25
has-miR-150-3p	miRNA	up	12	26
has-miR-181c-5p	miRNA	up	12	27
has-miR-4505	miRNA	up	12	30
has-miR-1254	miRNA	up	11	31
has-miR-140-3p	miRNA	up	11	33
has-miR-2276-3p	miRNA	up	11	34
has-miR-641	miRNA	up	11	35
has-miR-30b-5p	miRNA	up	10	37
has-miR-4695-5p	miRNA	up	10	38
has-miR-486-3p	miRNA	up	10	40
has-miR-29b-3p	miRNA	down	32	1
has-miR-424-5p	miRNA	down	28	2
has-miR-29a-3p	miRNA	down	23	3
has-miR-138-5p	miRNA	down	21	5
has-miR-145-5p	miRNA	down	16	14
has-miR-34a-5p	miRNA	down	16	15
has-miR-199a-3p	miRNA	down	14	18
has-miR-199b-3p	miRNA	down	14	19
has-miR-4269	miRNA	down	14	21
has-miR-493-5p	miRNA	down	14	22
has-miR-19a-3p	miRNA	down	13	23
has-miR-199b-5p	miRNA	down	12	28
has-miR-221-3p	miRNA	down	12	29
has-miR-125b-5p	miRNA	down	11	32
has-miR-1294	miRNA	down	10	36
has-miR-4773	miRNA	down	10	39
